# *EGFR*敏感突变NSCLC患者的最佳治疗模式探讨

**DOI:** 10.3779/j.issn.1009-3419.2015.02.11

**Published:** 2015-02-20

**Authors:** 钟 史, 云 范

**Affiliations:** 310022 杭州，浙江省肿瘤医院化疗中心 Department of Medical Oncology, Zhejiang Provincial Cancer Hospital, Hangzhou 310027, China

**Keywords:** 肺肿瘤, 表皮生长因子酪氨酸激酶抑制剂, 化疗, 贝伐珠单抗, Lung neoplasms, EGFR-TKI, Chemotherapy, Bevacizumab

## Abstract

目前表皮生长因子酪氨酸激酶抑制剂（epidermal growth factor receptor tyrosine kinase inhibitor, EGFR-TKI）已广泛应用于非小细胞肺癌（non-small cell lung cancer, NSCLC）患者，但如何将EGFR-TKI与化疗及其他靶向药物结合仍存在诸多争议。本综述阐述了EGFR-TKI与化疗的3种不同联合模式：前后序贯治疗、同步联合治疗及间插联合治疗; 分析EGFR-TKI与贝伐珠单抗联合治疗的优劣性; 初步探讨了EGFR突变患者的最佳治疗模式。

## Abstract (en)

Although epidermal growth factor receptor tyrosine kinase inhibitors (EGFR-TKIs) have been widely used in non-small cell lung cancer (NSCLC) patients, it is still controversial about how to combine EGFR-TKI with chemotherapy and other targeted drugs. We have made a summary on the current therapeutic models of EGFR-TKI combined with chemotherapy/bevacizumab in this review and aimed to find the optimal therapeutic strategy for NSCLC patients with EGFR mutation.

晚期非小细胞肺癌（non-small cell lung cancer, NSCLC）已进入分子分型时代，根据不同的分子特征来选择相应的分子靶向药物治疗，患者的中位生存时间有望达到3.5年^[1]^。表皮生长因子受体（epidermal growth factor receptor, *EGFR*）突变是目前研究最成熟、亚裔人群最常见的基因突变，亦是EGFR酪氨酸激酶抑制剂（EGFR tyrosine kinase inhibitor, EGFR-TKI）的疗效预测因素^[2]^。尽管EGFR-TKI已广泛应用于临床，如何更好地应用EGFR-TKI，如何将EGFR-TKI与化疗及其他靶向药物相结合仍存在诸多争议。本综述阐述EGFR-TKI与化疗的3种不同联合模式：前后序贯治疗、同步联合治疗及间插联合治疗; 分析EGFR-TKI与贝伐珠单抗联合治疗的优劣性; 探讨*EGFR*突变患者的最佳治疗模式。

## 1 EGFR-TKI与化疗的序贯治疗模式

即一种治疗方案失败后再序贯另一种治疗方案，这里主要的争议焦点在于如何选择一线治疗药物?如果一线选择化疗，什么时候用EGFR-TKI?维持治疗还是二线治疗？

### 1.1 一线治疗方案的选择：EGFR-TKI还是化疗？

*EGFR*突变的发现开启了EGFR-TKI的研究之门^[3, 4]^。其中最需要解决的一个重要问题是在*EGFR*突变的晚期NSCLC患者，一线TKI与一线化疗相比是否有疗效上的差异? IPASS研究是在非吸烟或轻度吸烟的腺癌患者中随机比较一线吉非替尼与一线的紫杉醇联合卡铂方案化疗^[2]^，主要观察目标为无进展生存期（progression free survival, PFS），在261例*EGFR*突变患者中，吉非替尼组的缓解率明显高于化疗组（71.2% *vs* 47.3%, *P* < 0.001），PFS延长（9.5个月*vs* 6.3个月，*P* < 0.001）。IPASS研究第一个在*EGFR*突变患者中证实一线TKI治疗与一线化疗相比有更长的PFS、更高的缓解率; 此后的类似研究，NEJ002、WJTOG3405、OPTIMAL、EURTAC、LUX-LUNG 3及LUX-LUNG 6等Ⅲ期随机对照研究均重复出同样结果^[5-10]^（表 1）; 无论是第一代可逆的或是第二代不可逆的EGFR-TKI，当其一线治疗*EGFR*突变患者时有效率高达55%-82.9%，PFS长达9.2个月-13.1个月，与一线含铂的两药化疗相比，PFS和客观缓解率（objective response rate, ORR）均显著提高。毫无疑问，这些结果奠定了EGFR-TKI在*EGFR*敏感突变患者中一线治疗的地位，成为此类患者首选的治疗方案; 近年，各权威指南均做出了相应的治疗推荐。

**1:** *EGFR*突变患者一线EGFR-TKI与化疗的Ⅲ期随机对照研究 Rrandomized phase Ⅲ trials comparing EGFR-TKI with chemotherapy as the first-line treatment for NSCLC patients with sensitive *EGFR* mutations

Drug name	Study	Investigator	Intervention	*n*	ORR (%)	*P*	PFS (mo)	*P*	OS (mo)	*P*
Gefitinib	IPASS M+	Mork *et al*^[2, 23]^	Gefitinib Paclitaxel/Carboplatin	132 129	71 47	< 0.001	9.5 6.3		21.6 21.9	0.990
	NEJ002	Maemondo *et al*^[5, 24]^	Gefitinib Paclitaxel/Carboplatin	114 110	73.7 30.7	< 0.001	10.8 5.4	< 0.001	27.7 26.6	0.483
	WJTOG3405	Mitsudomi *et all*^[6, 25]^	Gefitinib Docetaxel/Cisplatin	86 96	62.1 32.2	< 0.001	9.2 6.3	< 0.000, 1	35.5 38.8	0.443
Erlotinib	OPTIMAL	Zhou *et all*[7.26]	Erlotinib Gemcitabine/Carboplatin	82 72	82.9 36.1	< 0.001	13.1 4.6	0.000, 1	22.7 28.9	0.685
	EURTAC	Rosell *et al*^[8]^	Erlotinib Platinum based chemotherapy	86 87	55 11	< 0.001	9.7 5.2	< 0.000, 1	N/R	
Afatinib	LUX-LUNG 3	Yang *et al*^[9]^	Afatinib Pemetrexed/Cisplatin	230 115	56.1 22.6	< 0.001	11.1 6.9	< 0.001	N/R	
	LUX-LUNG 6	Wu *et al*^[10]^	Afatinib Pemetrexed/Cisplatin	242 122	66.9 23.0	< 0.000, 1	11.0 5.6	< 0.000, 1	N/R	
ORR: objective response rate; PFS: progression free survival; OS: overall survival; N/R: not reported; EGFR: epidermal growth factor receptor; TKI: tyrosine kinase inhibitor; NSCLC: non-small cell lung cancer.										

一线选择EGFR-TKI治疗的优势，除了ORR及PFS的提高外，还体现在对患者生活质量的改善及对疾病症状的快速缓解。前面7项随机对照研究中有4项研究进行了健康相关生活质量分析（health-related quality of life, QOL）。IPASS研究中，采用癌症治疗功能评估-肺癌问卷（Functional Assessment of Cancer Therapy - Lung Version, FACT-L）进行生活质量分析^[11]^，结果表明，在*EGFR*突变人群，吉非替尼治疗组的生活质量明显好于化疗组，（FACT-L评分吉非替尼组和化疗组生活质量提高患者的比例分别为：70.2% *vs* 44.5%，*P* < 0.001）; 其他生活质量评估表，试验结局指数（Trail Outcome Index, TOI）及肺癌亚量表（Lung Cancer Subscale, LCS）评估亦显示出同样结果（吉非替尼组和化疗组生活质量提高患者的比例分别为：TOI：70.2% *vs* 38.3%，*P* < 0.001;LCS：75.6% *vs* 53.9%，*P* < 0.001）; 而且，根据FACT-L评估，无论是*EGFR*突变患者还是全组患者，吉非替尼组的中位至生活质量恶化时间均明显长于化疗组。OPTIMAL研究和NEJ002研究的生活质量分析结果基本一致^[12, 13]^;在NEJ002研究^[13]^中，研究者还分析了中位至症状恶化时间（疼痛、咳嗽、日常功能），同样有利于EGFR-TKI组。LUX-LUNG3研究^[14]^的生活质量分析亦显示在*EGFR*突变患者，阿法替尼组比化疗组有更好的症状控制及生活质量。不难理解，由于EGFR-TKI的有效率高、副作用较小，与化疗相比，在应用EGFR-TKI治疗阶段患者有较好的生活质量。至于特殊人群，如不能耐受化疗的高龄患者、体能状态较差者及脑转移患者，EGFR-TKI治疗无疑更占优势^[15-22]^。

但遗憾的是，至今没有一项大型随机对照研究能证明一线应用EGFR-TKI相比一线选择化疗有生存上的优势。由于后续治疗有很高的交叉，如在IPASS研究中，化疗组64.3%的患者在疾病进展时接受了EGFR-TKI治疗，导致两组之间的总体生存期（overall survival, OS）无明显差异（21.6个月*vs* 21.9个月，*P*=0.99）^[23]^;上面提到的其他三项随机对照研究^[24-26]^，OS亦无明显差异。回顾性的研究^[27]^同样证实厄洛替尼的一线应用与二、三线应用不影响患者OS。这就意味着只要*EGFR*突变患者有机会接受EGFR-TKI治疗和化疗，无论采用什么治疗顺序似乎并不影响患者最后的生存结局。是否这是唯一的结果？2014年美国临床肿瘤学会（American Society of Clinical Oncology, ASCO）年会上发表了LUX-LUNG 3和LUX-LUNG 6的合并分析，共631例患者，结果显示全组患者阿法替尼一线治疗相比化疗有更长的OS时间（27.3个月*vs* 24.3个月，*P*=0.037, 4，HR=0.81），但这种OS的差异主要体现在*EGFR* 19外显子突变人群中（31.7个月*vs* 20.7个月，*P* < 0.000, 1），而在21外显子突变患者中一线化疗患者的OS略长于一线阿法替尼治疗（26.9个月*vs* 22.1个月，*P*=0.16）。是否在*EGFR* 19外显子突变人群，一线EGFR-TKI治疗的OS会长于一线化疗，需要进一步研究来验证。

### 1.2 EGFR-TKI的维持治疗

有关EGFR-TKI的维持治疗主要有两项Ⅲ期随机对照研究对*EGFR*突变患者进行了亚组分析。SATURN研究在欧洲进行^[28]^，共有883例晚期非选择NSCLC患者进入研究，与安慰剂相比，厄洛替尼的维持治疗延长了全组患者的PFS（12.3周*vs* 11.1周，HR=0.71，*P* < 0.000, 1）和OS（12.0个月 *vs* 11.0个月，*P*=0.008, 8）; *EGFR*突变患者的PFS改善最为明显（HR=0.1，*P* < 0.000, 1）。在中国进行的INFORM研究^[29]^中，吉非替尼的维持治疗得出类似结果; 最大获益人群仍在*EGFR*突变患者（HR=0.17）。对*EGFR*突变患者来说，EGFR-TKI治疗的疗效自然毋庸置疑。关键是，如果患者一线选择了化疗，EGFR-TKI的最佳介入时机在哪里？马上转换、维持治疗还是二线治疗？目前尚无随机对照研究来解决此问题。但需要注意的是，当患者经过一线化疗，如果仍有较高的肿瘤负荷和/或较明显的疾病症状时，毫无疑问，及时选择EGFR-TKI治疗是明智的决策。

## 2 化疗与EGFR-TKI的同步联合模式

### 2.1 临床研究

早期由于不清楚EGFR-TKI的确切靶向人群，EGFR-TKI与化疗的同步联合治疗模式主要是在非选择人群中进行研究。在2004年起的3年间，相继报道了四项在欧美进行的大型Ⅲ期随机对照研究（INTACT 12、INTACT 2、TRIBUTE及TALENT研究）^[30-33]^，比较吉非替尼（或厄洛替尼）与安慰剂同步联合含铂两药化疗方案一线治疗晚期NSCLC。研究对象为未经选择的晚期NSCLC患者，总人数超过4, 000例，结果没有一项研究显示在一线化疗的基础之上同步联合EGFR-TKI能改善生存。如何解释这些阴性结果？多数专家认为可能原因有两个，其一：化疗与EGFR-TKI的同步联合应用可能存在拮抗作用^[33]^;其二：治疗人群未经选择^[34, 35]^。那么，在选择人群中进行化疗同步联合EGFR-TKI治疗，结果又会如何？一项Ⅱ期的随机对照研究（CALGB30406）比较厄洛替尼单药与厄洛替尼同步联合卡铂紫杉醇方案治疗轻度/或不吸烟、初治、晚期肺腺癌患者182例^[36]^，两种治疗方案在近期疗效及生存上均无统计学差异; 亚组分析发现，*EGFR*突变患者无论采用何种治疗方式，其疗效均明显好于*EGFR*野生型患者; 在*EGFR*突变患者中，厄洛替尼联合卡铂/紫杉醇方案组的OS相对于厄洛替尼单药组似乎有延长的趋势（OS：38.1个月*vs* 27.6个月），但样本量偏小。至目前为止，尚无针对*EGFR*突变患者的随机对照研究，来比较一线EGFR-TKI单药治疗与化疗同步联合EGFR-TKI治疗; 鉴于在非选择人群中的阴性研究结果，不推荐化疗与EGFR-TKI的同步联合模式。

在一些特定人群，如EGFR-TKI获得性耐药患者，有研究表明，继续TKI治疗同步联合化疗能让一部分患者获益。Goldberg等^[37]^报道了一项回顾性研究，比较TKI获得性耐药患者的挽救治疗方案，34例患者继续接受厄洛替尼治疗同步联合化疗，44例患者进行单纯化疗，前者有更高的ORR（41% *vs* 18%），但PFS和OS无明显差异。在TKI获得性耐药这种特定人群，肿瘤细胞可能存在异质性^[38]^，一部分细胞对EGFR-TKI出现了耐药，仍有一部分细胞保留着对EGFR-TKI的敏感性，化疗与TKI的同步联合治疗模式不失为部分患者的有效治疗选择。是否EGFR-TKI与化疗的联合比单纯的化疗更能控制疾病，仍有待Ⅲ期随机对照研究的结果。

### 2.2 临床前研究

大多数化疗药物主要通过引起DNA损伤、抑制DNA合成及修复达到抗瘤作用^[39]^;EGFR-TKI的作用则是把细胞阻滞在G_1_期^[40]^，这就可能影响后续的细胞毒药物的抗肿瘤效应。临床前研究亦证实EGFR-TKI与化疗药物的不同给药顺序对抗肿瘤的协同作用可能有重要影响。加利福尼亚大学Davis癌症中心在肺癌细胞株A549和Calu-1中进行厄洛替尼与多西紫杉醇联合作用的体外研究^[41]^，结果发现：如果先用多西紫杉醇再序贯厄洛替尼，相比单用多西紫杉醇，凋亡加强; 而相反顺序，凋亡作用减弱。Cheng等^[42]^在A549（*EGFR*野生型，*K-Ras*突变型）、H1975（*EGFR* L858R-T790M突变型）和PC9（*EGFR*外显子19缺失）三种细胞株中进行吉非替尼与紫杉醇联合作用的体外研究，三种细胞株均给予三种不同给药顺序的干预; 紫杉醇序贯吉非替尼、吉非替尼序贯紫杉醇以及两药同步给药; 结果基本重复了Davis癌症中心的研究结果，在紫杉醇序贯吉非替尼的给药方式中，三种细胞株均表现为协同作用; 而在吉非替尼序贯紫杉醇及同步给药模式中均表现为拮抗作用。Li等^[43]^报道在先予培美曲塞再序贯或同步联合厄洛替尼的给药方式中，无论在厄洛替尼的耐药细胞株或是敏感细胞株均获得了协同抗肿瘤作用; 而相反给药顺序，则表现为拮抗作用。无论可能的机理如何，这些临床前研究均提示当先用细胞毒药物再序贯EGFR-TKI时，两者可表现为协同作用; 当相反顺序或同步联合时，两者则表现为拮抗效应。这似乎可以解释临床上化疗及EGFR-TKI同步联合治疗的阴性结果; 亦为化疗间插EGFR-TKI的治疗模式提供了临床前证据。

## 3 化疗与EGFR-TKI的间插治疗模式：即在化疗周期的某一时间段间插应用EGFR-TKI

### 3.1 间插模式的一线应用

Davis癌症中心率先报道了化疗与厄洛替尼间插治疗的Ⅰ期研究^[44]^，入选46例实体瘤患者，其中包括16例NSCLC，分成两组，两组化疗方案均为培美曲塞，每21天1个疗程，A组厄洛替尼每周（第2天、第9天和第16天）给药，剂量从800 mg递增至1, 400 mg; B组厄洛替尼第2天-第16天给药，剂量从150 mg递增至250 mg; 所有NSCLC患者，厄洛替尼治疗持续3个月-16个月; 结果共有6例患者达到部分缓解（partial response, PR），其中4例为NSCLC患者。此后Mok教授等^[45]^于2009年报告了一项在154例亚裔、晚期NSCLC患者中进行的有关间插治疗的Ⅱ期随机对照研究（FAST-ACT），比较一线吉西他滨联合顺铂或卡铂序贯厄洛替尼（第15天-第28天给药）与同样的化疗方案序贯安慰剂治疗，每28天1个疗程; 6个疗程后，疾病控制的患者分别以厄洛替尼或安慰剂维持治疗至疾病进展; 主要研究目标为8周的疾病无进展率; 结果显示，两组的8周疾病无进展率及肿瘤缓解率均无明显差异，但间插治疗组的PFS显著延长（29.4周*vs* 23.4周，HR=0.47，*P*=0.000, 2），两组的OS亦无差异。受到PFS延长的鼓励，研究者随后开展了更大样本量的Ⅲ期随机对照研究（FAST ACT-2）^[46]^;治疗方案基本沿用Ⅱ期研究，共入组451例亚裔患者，结果表明间插治疗组较对照组明显延长了PFS（7.6个月*vs* 6.0个月，*P* < 0.000, 1），近期缓解率更高（42.9% *vs* 17.8%，*P* < 0.000, 1）; 更令人惊喜的是间插治疗组较对照组显著延长了OS（18.3个月*vs* 15.2个月，*P*=0.042）。同时，全组患者中有241例患者进行了EGFR检测：在*EGFR*突变患者中，间插治疗组较对照组仍有更好的PFS和OS（PFS：16.8个月*vs* 6.9个月，*P* < 0.000, 1;OS：31.4个月*vs* 20.6个月，*P*=0.009）; 而在*EGFR*野生型患者，两组的PFS和OS均无明显差异; 研究者认为化疗与厄洛替尼的间插治疗是*EGFR*突变患者及选择性的*EGFR*突变状态未知患者一种可行的一线治疗模式。FAST ACT-2研究结果告诉我们，化疗间插联合EGFR-TKI治疗会进一步改善*EGFR*突变患者的生存; 但是，问题的症结在于，这种模式是否比EGFR-TKI与化疗的序贯应用更能改善生存？因为在FAST ACT-2研究中，对照组为化疗序贯安慰剂，无法证明间插治疗模式较标准的序贯治疗模式更能改善患者生存。况且，在该研究中，*EGFR*突变患者的OS为31.4个月，与EGFR-TKI一线治疗的生存数据（19.3个月-35.5个月）相比，并无明显优势。另一个不容忽视的问题是间插治疗模式是否会影响二线药物的选择。有研究^[47, 48]^报道*EGFR*突变患者存在肿瘤异质性，以致造成对TKI治疗反应的不一致，化疗间插EGFR-TKI的治疗模式是否能解决这种肿瘤的异质现象，值得进一步深入研究。

### 3.2 间插模式的二线应用

EGFR-TKI与培美曲塞、多西他塞均是晚期NSCLC二线治疗的标准药物。是否化疗与EGFR-TKI的双重打击能改善患者生存？在2012年的欧洲肿瘤内科学会（European Society for Medical Oncology, ESMO）年会上有两项Ⅱ期研究涉及此方面。在NVALT-10研究^[49]^中，含铂方案治疗失败的IIIb期-Ⅳ期NSCLC患者分别随机接受化疗间插厄洛替尼治疗和厄洛替尼单药治疗：间插治疗组中厄洛替尼给药时间在化疗后的第2天-第16天; 鳞癌患者选择多西他塞化疗，非鳞癌患者给予培美曲塞化疗; 共231例患者入组，结果显示：间插治疗组和单药组的PFS分别为6.1个月和4.9个月，差异无统计学意义（*P*=0.10），间插治疗组的OS明显延长（7.8个月*vs* 5.5个月，*P*=0.02）; 这项研究虽进行了相关生物标记物分析，但未能明确间插联合治疗模式的真正获益人群。另一项相似研究（S103研究）^[50]^在240例非鳞癌患者中进行，结果表明间插治疗组与单药组相比，PFS明显延长，OS数据尚不成熟。从这两项研究结果来看，化疗间插EGFR-TKI的二线治疗模式会对某些患者带来一定程度的PFS或OS改善，但因未对特定人群进行研究分析，导致它们的临床参考意义非常有限。在二线治疗中，已有多项研究^[51-53]^结果显示，EGFR-TKI对*EGFR*野生型患者的PFS明显短于化疗; 化疗间插EGFR-TKI的治疗模式可能同样不适用于野生型患者的二线治疗。

### 3.3 特定人群的间插治疗模式

在EGFR-TKI获得性耐药的*EGFR*突变患者，有研究报道，化疗间插EGFR-TKI治疗能让一部分患者获益。Yoshimura等^[54]^报告了一项前瞻性、小样本的研究结果：在27例EGFR-TKI获得性耐药患者中，培美曲塞间插厄洛替尼或吉非替尼治疗（第2天-第16天用药），ORR为25.9%，疾病控制率（disease control rate, DCR）为77.8%，PFS为7.0个月，OS为11.4个月。目前一些小样本研究的初步结果显示，化疗与EGFR-TKI的同步或间插联合治疗模式适合部分EGFR-TKI获得性耐药人群。

## 4 贝伐珠单抗与EGFR-TKI联合治疗EGFR突变患者

一项临床前研究^[55]^显示：*EGFR*突变的细胞株相比野生型细胞株有更高的血管内皮生长因子（vascular endothelial growth factor, VEGF）表达，用EGFR-TKI抑制突变细胞株，VEGF的表达相应降低; 同时阻断VEGF/EGFR通路，具有协同抗肿瘤活性。一项Ⅲ期随机对照研究^[56]^（Beta研究）比较贝伐珠单抗联合厄洛替尼与厄洛替尼单药用于一线化疗失败后的晚期NSCLC：在意向治疗（intent to treat, ITT）人群两组间未见OS差异，但在*EGFR*突变阳性的亚组，提示联合治疗OS有延长（HR=0.44）。2014年ASCO会议上报道了厄洛替尼联合贝伐珠单抗一线治疗*EGFR*突变患者的随机Ⅱ期研究（JO25567研究）^[57]^：共入组152例患者，比较厄洛替尼联合贝伐珠单抗与单用厄洛替尼的疗效，结果显示，联合治疗显著延长患者的PFS（16.0个月*vs* 9.7个月，*P*=0.001, 5），明显改善DCR（99% *vs* 88%，*P*=0.017, 7）; 但相应地，联合治疗方案有更高的不良反应发生率，包括高血压、蛋白尿、出血等; 联合治疗组≥3级的不良事件发生率高达91%，明显高于单药组的53%;联合治疗组因不良反应停止贝伐珠单抗治疗的比例亦达41%;两组的OS数据尚不成熟，初步显示无明显差异。JO25567研究^[57]^表明在*EGFR*突变的晚期NSCLC患者，同步联合抗血管生存药物及EGFR-TKI，能明显延长患者的PFS，但不良反应有增加。是否这种联合治疗模式能成为*EGFR*突变患者的标准治疗，仍有待进一步研究来验证。

## 5 结语

EGFR-TKI与化疗的序贯应用是*EGFR*突变患者目前最常应用的治疗模式，一线选择EGFR-TKI，患者有更高的缓解率、更长的无进展生存时间、更好的生活质量; 所以优先推荐EGFR-TKI用于突变患者的一线治疗。化疗与TKI的同步联合模式未给患者带来生存获益，目前只限于部分EGFR-TKI获得性耐药的人群。化疗与EGFR-TKI的间插联合模式给*EGFR*突变患者带来新的希望和挑战，是否会克服*EGFR*突变的异质性，成为最佳治疗模式，仍需进一步的随机对照研究。贝伐珠单抗与EGFR-TKI的联合治疗模式能显著延长*EGFR*突变患者的PFS，能否转化为生存的延长，仍有待研究中生存数据的观察。随着*EGFR*突变患者治疗选择的多样化及生存时间的延长，如何进行全程管理是医生必须要考虑的问题。

## References

[1] Johnson BE, Kris MG, Berry LD (2013). A multicenter effort to identify driver mutations and employ targeted therapy in patients with lung adenocarcinomas: The Lung Cancer Mutation Consortium (LCMC). J Clin Oncol.

[2] Mok TS, Wu YL, Thongprasert S (2009). Gefitinib or carboplatin-paclitaxel in pulmonary adenocarcinoma. N Engl J Med.

[3] Lynch TJ, Bell DW, Sordella R (2004). Activating mutations in the epidermal growth factor receptor underlying responsiveness of non-small-cell lung cancer to gefitinib. N Engl J Med.

[4] Paez JG, Janne PA, Lee JC (2004). *EGFR* mutations in lung cancer: Correlation with clinical response to gefitinib therapy. Science.

[5] Maemondo M, Inoue A, Kobayashi K (2010). Gefitinib or chemotherapy for non-small-cell lung cancer with mutated *EGFR*. N Engl J Med.

[6] Mitsudomi T, Morita S, Yatabe Y (2010). Gefitinib versus cisplatin plus docetaxel in patients with non-small-cell lung cancer harbouring mutations of the epidermal growth factor receptor (WJTOG3405): An open label, randomised phase 3 trial. Lancet Oncol.

[7] Zhou C, Wu YL, Chen G (2011). Erlotinib versus chemotherapy as first-line treatment for patients with advanced *EGFR* mutation-positive non-small-cell lung cancer (OPTIMAL, CTONG-0802): A multicentre, open-label, randomised, phase 3 study. Lancet Oncol.

[8] Rosell R, Carcereny E, Gervais R (2012). Erlotinib versus standard chemotherapy as first-line treatment for European patients with advanced *EGFR* mutation-positive non-small-cell lung cancer (EURTAC): A multicentre, open-label, randomized phase 3 trial. Lancet Oncol.

[9] Sequist LV, Yang JC, Yamamoto N (2013). Phase Ⅲ study of afatinib or cisplatin plus pemetrexed in patients with metastatic lung adenocarcinoma with *EGFR* mutations. J Clin Oncol.

[10] Wu YL, Zhou C, Hu CP (2014). Afatinib versus cisplatin plus gemcitabine for first-line treatment of Asian patients with advanced non-small-cell lung cancer harbouring *EGFR* mutations (LUX-Lung 6): an open-label, randomised phase 3 trial. Lancet Oncol.

[11] Thongprasert S, Duffield E, Saijo N (2011). Health-related quality-of-life in a randomized phase Ⅲ first-line study of gefitinib versus carboplatin paclitaxel in clinically selected patients from Asia with advanced NSCLC (IPASS). J Thorac Oncol.

[12] Chen G, Feng J, Zhou C (2013). Quality of life (QoL) analyses from OPTIMAL (CTONG-0802), a phase Ⅲ, randomised, open-label study of first-line erlotinib versus chemotherapy in patients with advanced EGFR mutation-positive non-small-cell lung cancer (NSCLC). Ann Oncol.

[13] Oizumi S, Kobayashi K, Inoue A (2012). Quality of life with gefitinib in patients with *EGFR*-mutated non-small cell lung cancer: quality of life analysis of North East Japan Study Group 002 Trial. Oncologist.

[14] Yang JC, Hirsh V, Schuler M (2013). Symptom control and quality of life in LUX-Lung 3: A phase Ⅲ study of afatinib or cisplatin/pemetrexed in patients with advanced lung adenocarcinoma with *EGFR* mutations. J Clin Oncol.

[15] Inoue A, Kobayashi K, Usui K (2009). First-line gefitinib for patients with advanced non-small-cell lung cancer harboring epidermal growth factor receptor mutations without indication for chemotherapy. J Clin Oncol.

[16] Uruga H, Kishi K, Fujii T (2010). Efficacy of gefitinib for elderly patients with advanced nonsmall cell lung cancer harboring epidermal growth factor receptor gene mutations: A retrospective analysis. Intern Med.

[17] Asami K, Koizumi T, Hirai K (2011). Geftinib as first-line treatment in elderly epidermal growth factor receptor-mutated patients with advanced lung adenocarcinoma: Results of a Nagano Lung Cancer Research Group Study. Clin Lung Cancer.

[18] Porta R, Sánchez-Torres JM, Paz-Ares L (2011). Brain metastases from lung cancer responding to erlotinib: The importance of EGFR mutation. Eur Respir J.

[19] Eichler AF, Kahle KT, Wang DL (2010). *EGFR* mutation status and survival after diagnosis of brain metastasis in non small cell lung cancer. Neuro Oncol.

[20] Park SJ, Kim HT, Lee DH (2012). Efficacy of epidermal growth factor receptor tyrosine kinase inhibitors for brain metastasis in non-small cell lung cancer patients harboring either exon 19 or 21 mutation. Lung Cancer.

[21] Wu YL, Zhou C, Cheng Y (2013). Erlotinib as second-line treatment in patients with advanced non-small-cell lung cancer and asymptomatic brain metastases: a phase Ⅱ study (CTONG-0803). Ann Oncol.

[22] Welsh JW, Komaki R, Amini A (2013). Phase Ⅱ trial of erlotinib plus concurrent whole-brain radiation therapy for patients with brain metastases from non-small-cell lung cancer. J Clin Oncol.

[23] Fukuoka M, Wu YL, Thongprasert S (2011). Biomarker analyses and final overall survival results from a phase Ⅲ, randomized, open-label, first-line study of gefitinib versus carboplatin/paclitaxel in clinically selected patients with advanced non-small cell lung cancer in Asia (IPASS). J Clin Oncol.

[24] Inoue A, Kobayashi K, Maemondo M (2013). Updated overall survival results from a randomized phase Ⅲ trial comparing gefitinib with carboplatin-paclitaxel for chemo-naive non-small cell lung cancer with sensitive *EGFR* gene mutations (NEJ002). Ann Oncol.

[25] Yoshioka H, Mitsudomi T, Morita S (2014). Final overall survival results of WJTOG 3405, a randomized phase 3 trial comparing gefitinib (G) with cisplatin plus docetaxel (CD) as the first-line treatment for patients with non-small cell lung cancer (NSCLC) harboring mutations of the epidermal growth factor receptor (*EGFR*). J Clin Oncol.

[26] Zhou C, Wu LY, Liu XQ (2012). Overall survival (OS) results from OPTIMAL (CTONG0802), a phase Ⅲ trial of erlotinib (E) versus carboplatin plus gemcitabine (GC) as first-line treatment for Chinese patients with *EGFR* mutation-positive advanced non-small cell lung cancer (NSCLC). J Clin Oncol.

[27] Rosell R, Moran T, Queralt C (2009). Screening for epidermal growth factor receptor mutations in lung cancer. N Engl J Med.

[28] Cappuzzo F, Ciuleanu T, Stelmakh L (2010). Erlotinib as maintenance treatment in advanced non-small-cell lung cancer: a multicentre, randomised, placebo-controlled phase 3 study. Lancet Oncol.

[29] Zhang L, Ma S, Song X (2012). Gefitinib versus placebo as maintenance therapy in patients with locally advanced or metastatic non-small-cell lung cancer (INFORM; C-TONG 0804): a multicentre, double-blind randomised phase 3 trial. Lancet Oncol.

[30] Giaccone G, Herbst RS, Manegold C (2004). Gefitinib in combination with gemcitabine and cisplatin in advanced non-small-cell lung cancer: a phase Ⅲ trial - INTACT 1. J Clin Oncol.

[31] Herbst RS, Giaccone G, Schiller JH (2004). Gefitinib in combination with paclitaxel and carboplatin in advanced non-small-cell lung cancer: a phase Ⅲ trial - INTACT 2. J Clin Oncol.

[32] Gatzemeier U, Pluzanska A, Szczesna A (2007). Phase Ⅲ study of erlotinib in combination with cisplatin and gemcitabine in advanced non-small-cell lung cancer: the Tarceva Lung Cancer Investigation Trial. J Clin Oncol.

[33] Herbst RS, Prager D, Hermann R (2005). TRIBUTE: a phase Ⅲ trial of erlotinib hydrochloride (OSI-774) combined with carboplatin and paclitaxel chemotherapy in advanced non-small-cell lung cancer. J Clin Oncol.

[34] Li T, Lara PN Jr, Mack PC (2010). Intercalation of erlotinib and pemetrexed in the treatment of non-small cell lung cancer. Curr Drug Targets.

[35] Gandara DR, Davies AM, Gautschi O (2007). Epidermal growth factor receptor inhibitors plus chemotherapy in non-small-cell lung cancer: biologic rationale for combination strategies. Clin Lung Cancer.

[36] Janne PA, Wang X, Socinski MA (2012). Randomized phase Ⅱ trial of erlotinib alone or with carboplatin and paclitaxel in patients who were never or light former smokers with advanced lung adenocarcinoma: CALGB 30406 trial. J Clin Oncol.

[37] Goldberg SB, Oxnard GR, Digumarthy S (2012). Chemotherapy with erlotinib or chemotherapy alone in advanced NSCLC with acquired resistance to EGFR tyrosine kinase inhibitors (TKI). J Clin Oncol.

[38] Oxnard GR, Arcila ME, Sima CS (2011). Acquired resistance to *EGFR* tyrosine kinase inhibitors in EGFR-mutant lung cancer: distinct natural history of patients with tumors harboring the T790M mutation. Clin Cancer Res.

[39] Gautschi O, Mack PC, Davies AM (2008). Pharmacogenomic approaches to individualizing chemotherapy for non-small-cell lung cancer: current status and new directions. Clin Lung Cancer.

[40] Normanno N, Bianco C, De LA (2003). Target-based agents against ErbB receptors and their ligands: a novel approach to cancer treatment. Endocr Relat Cancer.

[41] Mahaffey CM, Davies AM, Lara Jr PN (2007). Schedule-dependent apoptosis in *K-ras* mutant non-small-cell lung cancer cell lines treated with docetaxel and erlotinib: rationale for pharmacodynamic separation. Clin Lung Cancer.

[42] Cheng H, An SJ, Zhang XC (2011). *In vitro* sequence-dependent synergism between paclitaxel and gefitinib in human lung cancer cell lines. Cancer Chemother Pharmacol.

[43] Li T, Ling YH, Goldman ID (2007). Schedule-dependent cytotoxic synergism of pemetrexed and erlotinib in human non-small cell lung cancer cells. Clin Cancer Res.

[44] Davies AM, Ho C, Beckett L (2009). Intermittent erlotinib in combination with pemetrexed:phase Ⅰ schedules designed to achieve pharmacodynamic separation. J Thorac Oncol.

[45] Mok TS, Wu YL, Yu CJ (2009). Randomized, placebo-controlled, phase Ⅱ study of sequential erlotinib and chemotherapy as first-line treatment for advanced non-small-cell lung cancer. J Clin Oncol.

[46] Wu YL, Lee JS, Thongprasert S (2013). Intercalated combination of chemotherapy and erlotinib for patients with advanced stage non-small-cell lung cancer (FASTACT-2): a randomised, double-blind trial. Lancet Oncol.

[47] Chen ZY, Zhong WZ, Zhang XC (2012). *EGFR* mutation heterogeneity and the mixed response to EGFR tyrosine kinase inhibitors of lung adenocarcinomas. Oncologist.

[48] Zhou Q, Zhang XC, Chen ZH (2011). Relative abundance of *EGFR* mutations predicts benefit from gefitinib treatment for advanced non-small-cell lung cancer. J Clin Oncol.

[49] Aerts JG, Codrington H, Lankheet NA (2013). A randomized phase Ⅱ study comparing erlotinib versus erlotinib with alternating chemotherapy in relapsed non-small-cell lung cancer patients: the NVALT-10 study. Ann Oncol.

[50] Lee DH, Lee JS, Kim SW (2013). Three-arm randomised controlled phase 2 study comparing pemetrexed and erlotinib to either pemetrexed or erlotinib alone as second-line treatment for never-smokers with non-squamous non-small cell lung cancer. Eur J Cancer.

[51] Garassino MC, Martelli O, Broggini M (2013). Erlotinib versus docetaxel as second-line treatment of patients with advanced non-small-cell lung cancer and wild-type *EGFR* tumours (TAILOR): a randomised controlled trial. Lancet Oncol.

[52] Zhou Q, Cheng Y, Yang JJ (2014). Pemetrexed versus gefitinib as a second-line treatment in advanced non-squamous non-small-cell lung cancer patients harbouring wild-type *EGFR* (CTONG0806): a multicenter randomized trial. Ann Oncol.

[53] Okano Y, Ando M, Asami K (2013). Randomized phase Ⅲ trial of erlotinib (E) versus docetaxel (D) as second- or third-line therapy in patients with advanced non-small cell lung cancer (NSCLC) who have wild-type or mutant epidermal growth factor receptor (*EGFR*): Docetaxel and Erlotinib Lung Cancer Trial (DELTA). J Clin Oncol.

[54] Yoshimura N, Okishio K, Mitsuoka S (2013). Prospective assessment of continuation of erlotinib or gefitinib in patients with acquired resistance to erlotinib or gefitinib followed by the addition of pemetrexed. J Thorac Oncol.

[55] Van Cruijsen H, Giaccone G, Hoekman K (2005). Epidermal growth factor receptor and angiogenesis: Opportunities for combined anticancer strategies. Int J Cancer.

[56] Herbst RS, Ansari R, Bustin F (2011). Efficacy of bevacizumab plus erlotinib versus erlotinib alone in advanced non-small-cell lung cancer after failure of standard first-line chemotherapy (BeTa): a double-blind, placebo-controlled, phase 3 trial. Lancet.

[57] Seto T, Kato T, Nishio M (2014). Erlotinib alone or with bevacizumab as first-line therapy in patients with advanced non-squamous non-small-cell lung cancer harbouring *EGFR* mutations (JO25567): an open-label, randomised, multicentre, phase 2 study. Lancet Oncol.

